# Antiurolithiatic Potential of a Series of Phthalimide Derivatives on Calcium Oxalate Crystals

**DOI:** 10.1002/cbdv.202502823

**Published:** 2026-01-08

**Authors:** Daniele Regina Sonza, Laura Von Borell du Vernay França, Rita de Cássia Vilhena da Silva, Anelize Dada, Mariana Zanovello, Thaise Boeing, Priscila de Souza, Rogério Correa

**Affiliations:** ^1^ Postgraduate Program in Pharmaceutical Sciences, Biological activity of natural and synthetic products University of Vale do Itajaí Itajaí Brazil

**Keywords:** calcium oxalate crystals, isoindoline‐1,3‐dione derivatives, synthesis, urinary stones

## Abstract

Urolithiasis, primarily caused by calcium oxalate crystals, represents a major health concern due to high recurrence rates and limitations of current treatments in dissolving existing stones. This study investigated the antiurolithiatic potential of 15 phthalimide derivatives against calcium oxalate monohydrate (COM) and dihydrate (COD) crystals. The compounds were synthesized via condensation of phthalic anhydride with nitrogenous bases and evaluated using urine samples from rats with sodium oxalate‐induced crystallization. All derivatives reduced COM crystal formation at one or more concentrations, though only LD‐F 03 inhibited COD crystals. Notably, LD‐F 10 decreased COM by 89%, whereas LD‐F 11 achieved 92% COM inhibition. These results demonstrate the structural versatility of phthalimides and the need to optimize molecular design for selective activity. Overall, phthalimide derivatives show promising antiurolithiatic effects, warranting further studies on pharmacokinetics, safety, and potential combination with current therapies to reduce recurrence and surgical interventions.

## Graphical abstract

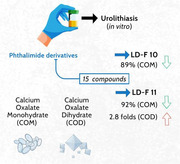

## 1 Introduction

Urolithiasis refers to the formation of urinary stones anywhere in the urinary tract. The stones are chemically composed of inorganic and organic crystals. However, in humans, 60%–85% are calcium derivative stones, of which calcium oxalate is prevalent [1, 2]. The lifetime risk of developing urinary stones (calculi) is about 12% for men and 6% for women [3], and recurrence is expected in 50% of patients over 5–10 years [1, 4].

In most patients, the etiology combines several factors, such as environmental, dietary, hormonal, and genetic components [2]. The formation of the urinary calculi occurs when there is a deficiency of stone‐inhibiting factors and supersaturated urine due to low urinary volume and/or hypercalciuria, hyperuricosuria, increased amounts of citrate, magnesium, and glycosaminoglycans [3]. This leads to crystal formation, growth, aggregation, and retention within the kidneys, mainly in the renal calyces and pelvis, where they can be found free or attached to the renal papillae [4]. Pieces of evidence suggest that there are several genes involved in renal tubular handling of lithogenic substrates and inhibitors of crystallization in stone disease in the general population [2]. Moreover, obesity, diabetes, hypertension, and metabolic syndrome are considered risk factors for stone formation, which, in turn, can lead to hypertension, chronic kidney disease (CKD), and end‐stage renal disease [4].

The management of patients with symptomatic urolithiasis is based on surgical approaches, of which the success depends on the physician's experience, the size, location, and composition of the stone, and the patient's characteristics, such as medical comorbidities and anatomy [4]. Furthermore, nutritional advice for patients with calcium stones includes increasing water intake and maintaining a balanced diet, while drug treatment may be considered if stones continue to recur despite the above measures, or if the risks of CKD and/or metabolic bone disease are considerable, or in those who have severe metabolic abnormalities in the urine. For this, the drugs used are thiazides, which reduce calciuria and might improve bone mineral density [5]. Moreover, allopurinol or febuxostat could be useful in patients with calcium stones who are hyperuricosuric [6]. Although prevention of new calcium stones is possible, there is no pharmacological treatment that can dissolve existing calcium stones [4]. In this way, the development of molecules with antiurolithiatic potential could improve the management of urinary stones, decreasing the need for surgical procedures, improving the success rates of stone removal, and even decreasing the chances of recurrence.

In this regard, imides are potential compounds to be explored. They are a group of heterocyclic organic compounds characterized by the functional group ─CO‐N(R)‐CO─, which is a privileged structure that has been widely used as an effective model for drug discovery. These groups are connected through a carbon chain, typically forming a cyclic structure with fewer than seven carbon atoms. Notably, this class of heterocycles is also represented in Brazilian folk medicine: imide‐containing compounds have been identified in the aerial parts of *Phyllanthus sellowianus*, popularly known as “quebra‐pedra,” a medicinal plant widely distributed in the flora of Santa Catarina. *Quebra‐pedra* is traditionally used for the treatment and prevention of kidney stones, with several studies supporting its nephroprotective and antiurolithiatic properties [7]. Consequently, the interest in cyclic imides was strengthened, culminating in the discovery and isolation of the natural alkaloid phyllanthimide [8]. This finding not only highlighted the chemical relevance of this scaffold but also reinforced its potential pharmacological value in the context of urolithiasis. Considering that *Phyllanthus* species have long been recognized in traditional medicine for their ability to prevent and dissolve urinary stones, the presence of an imide‐containing alkaloid in this genus provides additional support for the hypothesis that imide‐based molecules may modulate crystal formation pathways. Building on this rationale, the present study aimed to synthesize a series of phthalimide derivatives and systematically evaluate their ability to inhibit the formation of urinary stones. By exploring structural variations within the imide framework, we sought to identify compounds capable of interfering with crystallization processes relevant to nephrolithiasis.

## 2 Materials and Methods

### 2.1 Synthesis

In this study, phthalimides were synthesized by reacting phthalic anhydride with nitrogenous bases in a solvent under reflux. Fifteen different compounds were obtained via a condensation reaction, utilizing equimolar amounts (10 mmol each) of commercially available phthalic anhydride and nitrogenous bases, such as substituted anilines and phenylhydrazines, with 15 mL of acetic acid as the solvent. The reaction mixture was refluxed for 2 h, after which 25 mL of cold distilled water was added to stop the reaction. The resulting products were filtered and recrystallized in ethanol. The nitrogenous bases employed included benzohydrazide (01), methoxybenzohydrazide (02), 2‐aminobenzothiazole (03, 04), p‐toluidine (05), 3,5‐dimethylaniline (06), 4‐ethylaniline (07), dinitrophenylhydrazine (08), 4‐aminopyridine (09), p‐anisidine (10), 4‐aminoacetophenone (11), 2‐aminothiophenol (12), 2‐phenylethylamine (13), 4‐chloroaniline (14), and benzylamine (15).

Reaction progress and completion were monitored using thin‐layer chromatography (TLC) on silica gel plates (0.2 mm thick with aluminum backing and F254 fluorescence indicator, Merck). A hexane‐ethyl acetate (1:1) mobile phase was used, and plates were analyzed under UV light. The synthesized compounds were characterized by melting point analysis (MP‐301 – Microquímica), Fourier‐transform infrared spectroscopy (FT‐IR) with ATR (Agilent Cary 630 FTIR), and nuclear magnetic resonance (NMR) spectroscopy for ^1^H (300 MHz) and ^13^C (75 MHz) using Bruker Avance DPT‐300, with samples dissolved in DMSO‐d6 or CDCl_3_. Mass spectra were recorded with Agilent LC‐MSMS 6420 using direct insertion (DIMS) with acetone‐diluted samples. All the compounds were given the prefix LD‐F, where L is with respect to the ligand term, D concerning the derivative term, and F to function. The detailed synthesis steps and characterization of the phthalimide derivatives, including their NMR spectra, are described in the Supporting Information.

### 2.2 Animals

Twenty‐four normotensive female Wistar rats, 3–4 months old, were used for urine sample collection. The animals were provided by Univali's vivarium and were maintained at controlled room temperature (22 ± 2°C), 12‐h light/dark cycle, with free access to food and water. The methodologies used to collect urine were previously described [9]. The protocol was submitted and approved by Univali's animal experimentation ethics committee, CEUA/Univali (n. 013/21). All the experiments were conducted under international standards and ethical guidelines on animal welfare.

### 2.3 Precipitation of Calcium Oxalate Crystals in the Urine

The rat's urine samples were divided into different aliquots of 500 µL (*n* = 6 each group): Vehicle group (VE: containing only urine), positive control group (containing urine and potassium citrate (C+ 10 mg/mL), and experimental compound groups (containing urine with the different phthalimide derivatives (LD‐F 01–15) at three different concentrations 0.1, 0.3, and 1 mg/mL). For the formation of urinary stones, precipitation of calcium oxalate (CaC_2_O_4_) was induced in each tube by adding 0.1 M sodium oxalate in the urine at 37°C (40 µL/mL). After 60 min of addition of sodium oxalate, the number of CaC_2_O_4_ crystals was evaluated using a Neubauer chamber under a microscope with 40x magnification and expressed as the number of crystals (monohydrate or dihydrate) per milliliter of urine [9].

### 2.4 Statistical Analysis

Statistical analysis was performed using GraphPad Prism version 7.00 for Windows (GraphPad Software, La Jolla, CA, USA). Results are presented as mean ± standard error of the mean (SEM). For all analyses, one‐way analysis of variance (ANOVA) was used, followed by Dunnett's multiple comparison test. *p*‐Values <0.05 were considered statistically significant.

## 3 Results

### 3.1 Characterization of Phthalimide Derivatives

The IUPAC name and molecular formula of the 15 compounds are described below. Further characterization details are summarized in the Supporting Information.

LD‐F 01: N‐(1,3‐dioxo‐1,3‐dihydro‐2H‐isoindol‐2‐yl)‐benzamide; C_15_H_10_N_2_O_3_. LD‐F 02: N‐(1,3‐dioxo‐1,3‐dihydro‐2H‐isoindol‐2‐yl)‐4‐methoxybenzamide; C_8_H_10_N_2_O_2_. LD‐F 03: 2‐(1,3‐thiazol‐2‐yl)‐1H‐isoindole‐1,3(2H)‐dione; C_11_H_6_N_2_O_2_S_2_. LD‐F 04: 2‐(1,3‐benzothiazol‐2‐yl)‐1H‐isoindole‐1,3(2H)‐dione; C_15_H_8_N_2_O_2_S. LD‐F 05: 2‐(4‐methylphenyl)‐1H‐isoindole‐1,3(2H)‐dione; C_15_H_11_NO_2_. LD‐F 06: 2‐(3,5‐dimethylphenyl)‐1H‐isoindole‐1,3(2H)‐dione; C_16_H_13_NO_2_. LD‐F 07: 2‐(4‐ethylphenyl)‐1H‐isoindole‐1,3(2H)‐dione; C_16_H_13_NO_2_. LD‐F 08: 2‐(2,4‐dinitrophenyl)isoindole‐1,3‐dione; C_14_H_7_N_3_O_6_. LD‐F 09: 2‐(pyridin‐4‐yl)‐1H‐isoindole‐1,3(2H)‐dione; C_13_H_8_N_2_O_2_. LD‐F 10: 2‐(4‐methoxyphenyl)‐1H‐isoindole‐1,3(2H)‐dione: C_15_H_11_NO_3_. LD‐F 11: 2‐(4‐acetylphenyl)‐1H‐isoindole‐1,3(2H)‐dione; C_16_H_11_NO_3_. LD‐F 12: 2‐(phenylsulfanyl)‐2,3‐dihydro‐1H‐isoindole‐1,3‐dione; C_14_H_9_NO_2_S. LD‐F 13: 2‐(2‐phenylethyl)‐1H‐isoindole‐1,3(2H)‐dione; C_16_H_13_NO_2_. LD‐F 14: 2‐(4‐chlorophenyl)‐1H‐isoindole‐1,3(2H)‐dione; C_16_H_13_NO_2_. LD‐F 15: 2‐benzyl‐1H‐isoindole‐1,3(2H)‐dione; C_15_H_11_NO_2_.

### 3.2 Inhibitory Effect of LD‐F 01–15 on Urinary Calcium Oxalate Crystals

**FIGURE 1:**
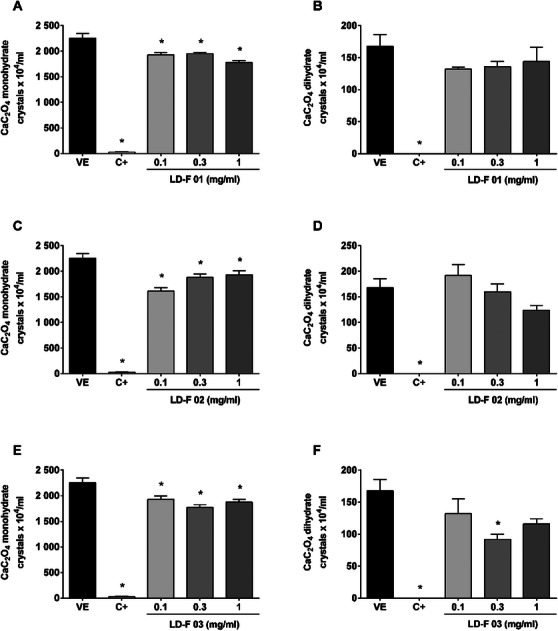
**Effect of LD‐F compounds 01, 02, and 03 on the formation of monohydrate and dihydrate calcium oxalate (CaC_2_O_4_) crystals**. (A and B) LD‐F 01; (C and D) LD‐F 02; (E and F) LD‐F 03. Results are expressed as mean ± SEM. Statistical analysis was performed by one‐way analysis of variance (ANOVA) followed by Dunnett's post‐test. *p < 0.05 when compared to the vehicle group (VE). C+: potassium citrate (10 mg/mL).

Figure 1 shows the effect of LD‐F 01, 02, and 03. As observed, the positive control (C+), potassium citrate, a well‐established reference treatment for preventing urinary stone formation, significantly reduced (98%) the number of urinary monohydrate CaC_2_O_4_ crystals compared to the vehicle group (2256 ± 115 crystals/mL), as well as completely inhibited the formation of dihydrate crystals compared to the vehicle (168 ± 23 crystals/mL). The experimental compounds LD‐F 01 and 02 significantly reduced the number of monohydrate urinary CaC_2_O_4_ crystals at all concentrations evaluated (0.1, 0.3, and 1 mg/mL); however, the formation of dihydrate crystals was not inhibited. LD‐F 03 decreased the formation of monohydrate CaC_2_O_4_ crystals at 14%, 21%, and 16% at 0.1, 0.3, and 1 mg/mL, respectively. The concentration of 0.3 mg/mL also reduced the number of dihydrate crystals (45%).

**FIGURE 2:**
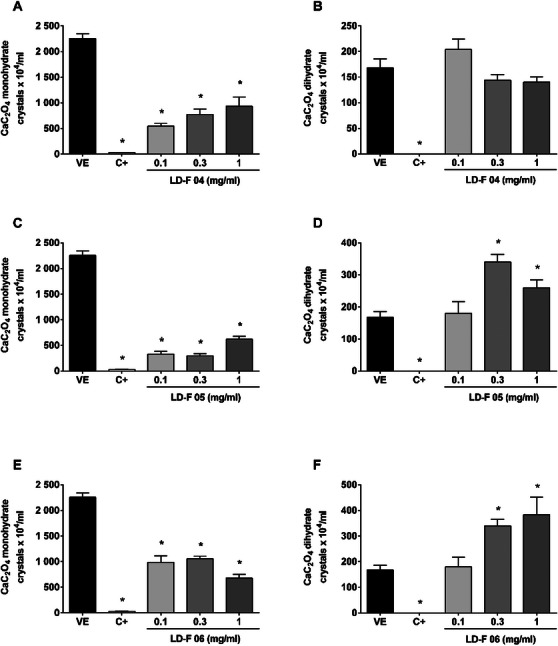
**Effect of LD‐F compounds 04, 05, and 06 on the formation of monohydrate and dihydrate calcium oxalate (CaC_2_O_4_) crystals**. (A and B) LD‐F 04; (C and D) LD‐F 05; (E and F) LD‐F 06. Results are expressed as mean ± SEM. Statistical analysis was performed by one‐way analysis of variance (ANOVA) followed by Dunnett's post‐test. *p < 0.05 when compared to the vehicle group (VE). C+: potassium citrate (10 mg/mL).

Figure 2 shows the effect of LD‐F 04, 05, and 06. LD‐F 04 reduced the number of monohydrate urinary CaC_2_O_4_ crystals from 75% to 58% at 0.3 and 1 mg/mL, respectively; however, the formation of dihydrate crystals was not inhibited. LD‐F 05 reached a maximum inhibition of 86% at 0.3 mg/mL, while LD‐F 05 achieved 69% inhibition at 1 mg/mL for monohydrate CaC_2_O_4_ crystals. In contrast, both compounds increased the number of dihydrated CaC_2_O_4_ crystals at 0.3 and 1 mg/mL, respectively, compared to the vehicle.

**FIGURE 3:**
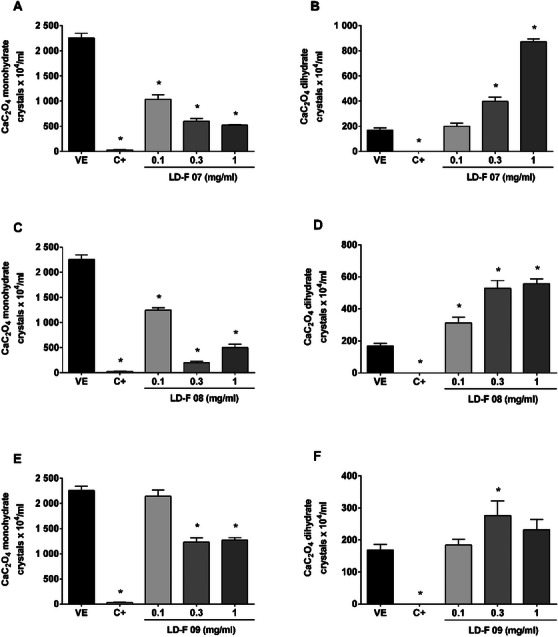
**Effect of LD‐F compounds 07, 08, and 09 on the formation of monohydrate and dihydrate calcium oxalate (CaC_2_O_4_) crystals**. (A and B) LD‐F 07; (C and D) LD‐F 08; (E and F) LD‐F 09. Results are expressed as mean ± SEM. Statistical analysis was performed by one‐way analysis of variance (ANOVA) followed by Dunnett's post‐test. *p < 0.05 when compared to the vehicle group (VE). C+: potassium citrate (10 mg/mL).

Figure 3 shows the effect of LD‐F 07, 08, and 09, which displayed a similar inhibition profile to compounds 05 and 06, reducing the number of monohydrate CaC_2_O_4_ crystals and increasing those dihydrates.

LD‐F 07 showed maximum inhibition of monohydrate urinary CaC_2_O_4_ crystals at 1 mg/mL (77%), LD‐F 08 and LD‐F 09 at 0.3 mg/mL (91% and 45%, respectively). In the same mentioned concentrations, the increase of dihydrate crystals reached values of 872 ± 23, 528 ± 48, and 276 ± 45 crystals/mL, respectively, while the vehicle presented only 168 ± 23 crystals/mL.

**FIGURE 4:**
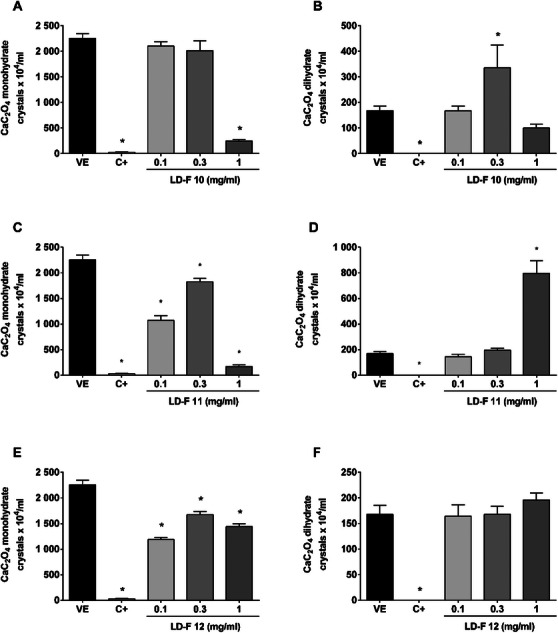
**Effect of LD‐F compounds 10, 11, and 12 on the formation of monohydrate and dihydrate calcium oxalate (CaC_2_O_4_) crystals**. (A and B) LD‐F 10; (C and D) LD‐F 11; (E and F) LD‐F 12. Results are expressed as mean ± SEM. Statistical analysis was performed by one‐way analysis of variance (ANOVA) followed by Dunnett's post‐test. *p < 0.05 when compared to the vehicle group (VE). C+: potassium citrate (10 mg/mL).

Figure 4 shows the effect of LD‐F 10, 11, and 12. LD‐F 10 in the lower concentrations did not inhibit the monohydrate crystals, while the 0.3 mg/mL concentration increased the dihydrate crystals. On the other hand, the bigger concentration decreased monohydrate crystals (89%) without changing the number of dihydrate CaC_2_O_4._

LF‐F 11 similarly decreased monohydrate crystals (92%) but increased dihydrates by 2.8‐fold. In turn, LDF 12 did not change the number of dihydrates CaC_2_O_4_ but decreased monohydrates by 47%.

**FIGURE 5:**
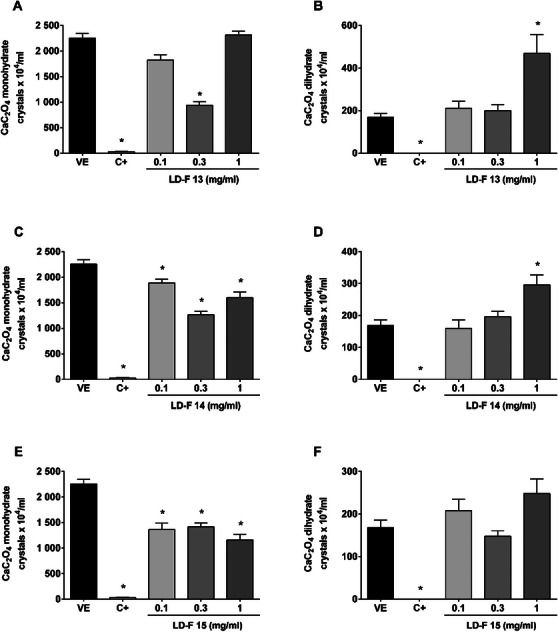
**Effect of LD‐F compounds 13, 14, and 15 on the formation of monohydrate and dihydrate calcium oxalate (CaC_2_O_4_) crystals**. (A and B) LD‐F 13; (C and D) LD‐F 14; (E and F) LD‐F 15. Results are expressed as mean ± SEM. Statistical analysis was performed by one‐way analysis of variance (ANOVA) followed by Dunnett's post‐test. *p < 0.05 when compared to the vehicle group (VE). C+: potassium citrate (10 mg/mL).

Finally, Figure 5 shows the effect of LD‐F 13, 14, and 15. LD‐F 13 decreased monohydrate CaC_2_O_4_ formation at 0.3 mg/mL (58%), but this concentration did not decrease the number of dihydrate crystals. By increasing the concentration to 1 mg/mL, the effect over monohydrates did not improve, and the number of dihydrate crystals was increased (2.8‐fold). LD‐F 14 also increased dihydrate crystals in the bigger concentration and decreased monohydrate crystals. LD‐F 15 presented similar values in decreasing monohydrate crystals, but the 1.4‐fold increase in dihydrate formation was not statistically significant.

## 4 Discussion

Phthalimides are part of the class of biologically active N‐heterocycles, which are found in pharmaceuticals, natural products, agrochemicals, polymers, and dyes, and also serve as building blocks in organic transformations [10]. They are a class of compounds with diverse chemistry and applications across various medicinal fields, including the urinary system. For instance, naltalimide, a compound that acts as a μ‐opioid receptor partial agonist, showed an inhibitory action on the micturition reflex, which would be a promising therapeutic option for overactive bladder [11, 12]. Additionally, the ability of certain phthalimide derivatives to modulate oxidative stress and inflammation might contribute to their protective role in conditions like nephrotoxicity or urinary tract disorders [13, 14].

Patients experiencing urinary stones can have symptoms such as pain in the back or lower abdomen, pain when urinating, and the presence of blood in the urine, nausea, and vomiting. In most of these patients, only dietary changes or stone‐expulsive therapies are not very successful, and intervention procedures are not always easily accessible [1]; however, there is no pharmacological treatment that can dissolve existing calcium stones [4], reinforcing the importance of the search for new bioactive molecules. In this context, phthalimides are an intriguing class of compounds in drug discovery due to their structural versatility and biological activities.

As mentioned before, supersaturation of urine, crystallization, and retention of stones within the urinary system are involved in the formation of urinary stones. Although there are different chemical types of stones, calcium stones are the most common and occur as calcium oxalate and calcium phosphate crystals, alone or in combination [4], which justifies the use of the CaC_2_O_4_ crystal precipitation model to investigate the effects of the phthalimide derivatives presented herein.

The CaC_2_O_4_ in kidney stones is presented as monohydrate (COM) or dihydrate (COD) crystals. COM crystals are prevalent; they are thin and acquire a ‘dumb‐bell’ or ‘oval’ shape through twinning, while COD crystals are tetragonal bipyramidal in shape [4, 15]. Thus, we have evaluated the effect of phthalimide derivatives in the formation of both forms. As presented in our results, the 15 phthalimide derivatives studied were able to decrease in one or more concentrations the number of COM crystals; nevertheless, only compound LD‐F 03 decreased COD crystals, though some compounds did not alter their formation, and others increased their number. Conversely, it is important to mention that COM crystals are more stable than COD and usually have more affinity and adhesion in the tubular epithelial cells than COD [15, 16].

Regarding COM, the compounds LD‐F 01‐03 have similar results, but the modifications made in the molecular structure improved their inhibitory effect. The molecule LD‐F 05, for instance, reached a bigger COM inhibition than LD‐F 01‐03, but increased COD. The same profile was seen for the other compounds. The biggest COM inhibition found was for LD‐F11 (92%), but this compound also induced the biggest COD increase. Analyzing the two types of crystals together, LD‐F 10 at 1 mg/mL showed the best antiurolithiatic effect, decreasing COM by 89% without changing the number of CODs. Regarding the chemical structure of these components, the 4‐acetylphenyl substituent of the LD‐F11 deserves attention. The presence of the carbonyl group at the para position relative to the aromatic ring allows for additional interactions, such as hydrogen bonding and π‐π interaction with aromatic residues in proteins. About LD‐F 10, the methoxy group in the para position of the phenolic ring is an electron donor through resonance effects, which can increase the electron density in the aromatic ring. This characteristic may influence the compound's affinity for biological targets, such as receptors or enzymes, by facilitating Van der Waals interactions or π‐π interactions. Furthermore, the methoxy group can serve as a binding site for hydrogen bonding, contributing to molecular recognition.

The positive control of the experiment, potassium citrate, decreased COM by 98% and COD by 100%. Indeed, randomized trials have shown the efficacy of citrate [17, 18]. It is indicated for patients with decreased urinary citrate excretion; nonetheless, it might increase the risk of forming calcium phosphate crystals because it raises urinary pH (via its metabolism to bicarbonate by the liver) [4].

The urinary crystallization model used in this study is widely recognized as an initial and reliable screening approach, as it enables a direct assessment of the ability of compounds to inhibit or modulate the formation of calcium oxalate crystals, which represent the main component of human kidney stones. This method offers early and biologically relevant information regarding anti‐crystallization potential and serves as an important step prior to conducting in vivo evaluations. However, while our study provides significant insights into the antiurolithiatic potential of phthalimide derivatives through in vitro assessments, it is crucial to recognize the limitations of this approach. The complexity of urolithiasis involves multifactorial physiological processes, including urine supersaturation, crystal nucleation, aggregation, and retention, all of which are influenced by dynamic biological factors that cannot be fully replicated in an in vitro setting. Future research should focus on screening phthalimide derivatives for inhibitory effects on stone‐forming processes, evaluating their pharmacokinetics and safety, and exploring their potential synergistic effects with existing treatments. These efforts could pave the way for novel therapies targeting urinary calculus.

It is worth noting that our previous study conducted the in‐silico analyses of these 15 cyclic imides, predicting their pharmacokinetic behavior and safety profiles. These computational evaluations provided insights into absorption, distribution, metabolism, excretion, and potential toxicological effects, including mutagenicity, hepatotoxicity, and cardiotoxicity. These analyses highlighted compounds 09, 12, 14, and potentially 01–04 as the most promising candidates. Besides, in functional assays, all evaluated derivatives exhibited relaxant effects on pre‐contracted jejunal segments. Among them, compounds 01, 02, 03, 04, 06, 13, and 15 showed marked inhibition of acetylcholine‐evoked contractions [19]. Nevertheless, these predictive tools have inherent limitations, particularly regarding the accuracy of absorption, metabolism, and toxicity estimates, which require validation in more complex biological systems. Future studies should therefore combine advanced computational modeling with in vitro and in vivo experiments, aiming to confirm pharmacokinetic and toxicological profiles and to support the rational development of these compounds as therapeutic candidates.

## 5 Conclusions

Phthalimides represent a promising class of compounds in the realm of drug discovery for urinary calculus due to their structural versatility and diverse biological activities. In the present study, the 15 phthalimide derivatives were effective in diminishing the number of crystal formations in the urine. Notably, compounds like LD‐F 10 demonstrated significant inhibitory activity on COM crystals without increasing COD formation, showcasing an advantageous profile. These findings emphasize the importance of tailoring molecular structures to optimize bioactivity. Compared to traditional treatments like potassium citrate, phthalimides offer a novel mechanism of action and may circumvent some limitations of current therapies. Future studies should further explore their pharmacokinetics, safety profiles, and potential synergies, paving the way for innovative strategies to manage urinary stone diseases.

## Author Contributions

Daniele Regina Sonza and Laura Von Borell du Vernay França were responsible for the synthesis of the compounds. Rita de Cássia Vilhena da Silva, Anelize Dada, Mariana Zanovello, and Thaise Boeing conducted the pharmacological experiments. Priscila de Souza, Thaise Boeing, and Rogério Correa contributed to data acquisition, manuscript drafting, and revision. All authors reviewed and approved the final version of the manuscript.

## Conflicts of Interest

The authors declare no conflicts of interest.

## Supporting information




**Supporting File 1**: cbdv70808‐sup‐0001‐SuppMat.pdf

## Data Availability

The data that support the findings of this study are available from the corresponding author upon reasonable request.
