# Supplementary material for: Antiurolithiatic Potential of a Series of Phthalimide Derivatives on Calcium Oxalate Crystals
Source: Chem Biodivers. 2026 Jan 8;23(1):e02823. doi: 10.1002/cbdv.202502823 (PMC12781043; doi:10.1002/cbdv.202502823)

SUPPLEMENTARY MATERIAL

Synthesis and characterization of phthalimide derivatives:

To synthesize N-(1,3-dioxo-1,3-dihydro-2H-isoindol-2-yl)benzamide (LD-F 01), 1.38 g of benzohydrazide and 1.48 g of phthalic anhydride were combined in 15 mL of glacial acetic acid and heated under reflux for 2 hours. After cooling in an ice-water bath, the precipitate was filtered, washed with cold water, dried in a vacuum desiccator for 24 hours, and recrystallized from hot ethanol, yielding pale yellow crystals.

The same procedure was employed for other derivatives:

Compound LD-F 02: Methoxybenzohydrazide (1.71 g) yielded white crystals.

Compound LD-F 03: 2-Aminothiazole (1.00 g) produced light brown crystals.

Compound LD-F 04: 2-Aminobenzothiazole (1.00 g) gave white crystals.

Compound LD-F 05: p-Toluidine (1.08 g) yielded white crystals.

Compound LD-F 06: 3,5-Dimethylaniline (1.23 g) formed light gray crystals.

Compound LD-F 07: 4-Ethylaniline (1.07 g) resulted in purple crystals.

Compound LD-F 08: 2,4-Dinitrophenylhydrazine (2.00 g) gave yellow crystals.

Compound LD-F 09: 4-Aminopyridine (2.00 g) produced white crystals with a very low yield, insufficient for FTIR analysis.

Compound LD-F 10: p-Anisidine (0.88 g) yielded green crystals.

Compound LD-F 11: 4-Aminoacetophenone (1.36 g) resulted in white crystals.

Compound LD-F 12: 2-Aminothiophenol (1.36 g) produced dark gray crystals.

Compound LD-F 13: 2-Phenylethylamine (1.22 g) gave white crystals.

Compound LD-F 14: 4-Chloroaniline (1.30 g) resulted in white crystals.

Compound LD-F 15: Benzylamine (1.08 g) yielded white crystals.

For all compounds, the reactions were monitored by thin-layer chromatography, and products were recrystallized as needed. The NMR spectra obtained for the synthesized compounds are also provided below.

N-(1,3-dioxo-1,3-dihydro-2H-isoindol-2-yl)benzamide (LD-F 01): Molecular formula: $C_{15}H_{10}N_2O_3$. Molar mass: 266.25 g/mol. Melting point (°C): 217; (theor. 218). FTIR (cm^{-1}): 1733.2 (C=O, imide); 1505.8 (N-H); 693.3 (C-H, aromatic ring). NMR - ^1H ($CDCl_3$) (ppm): 1.584 (chloroform water); 7.240 (chloroform); 7.427 -

7.935 (aromatic ring); 8.230 (NH). NMR - ^{13}C ($CDCl_3$) (ppm): 124.117 - 134.794 (aromatic ring); 164.960 (C=O, imide).

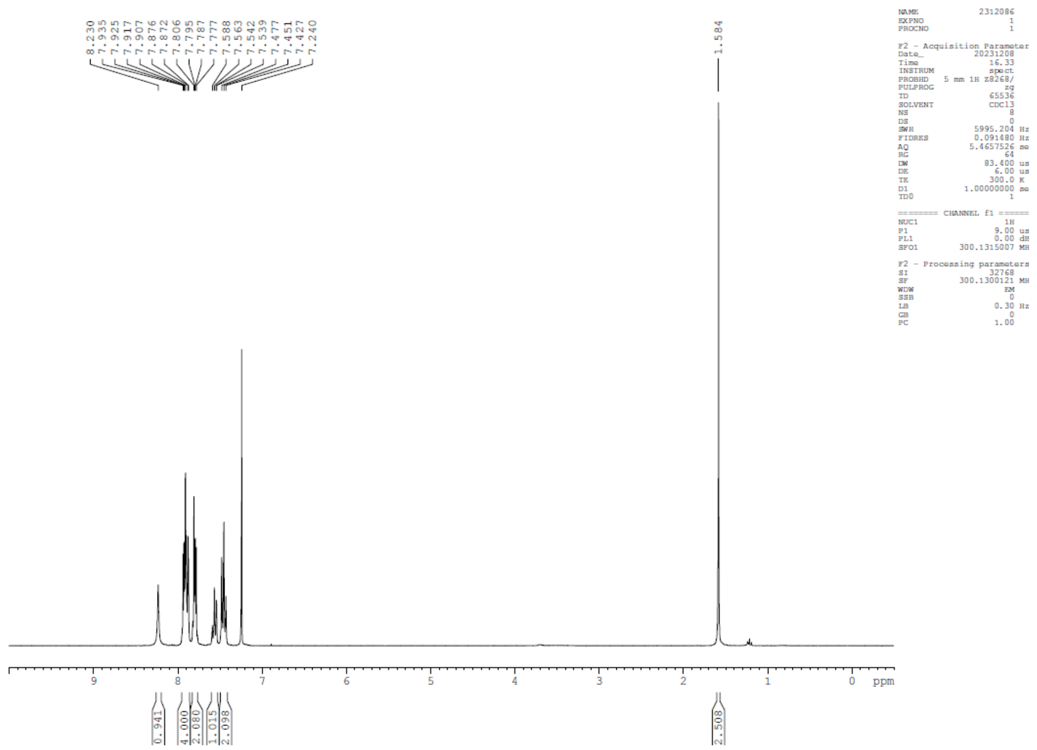

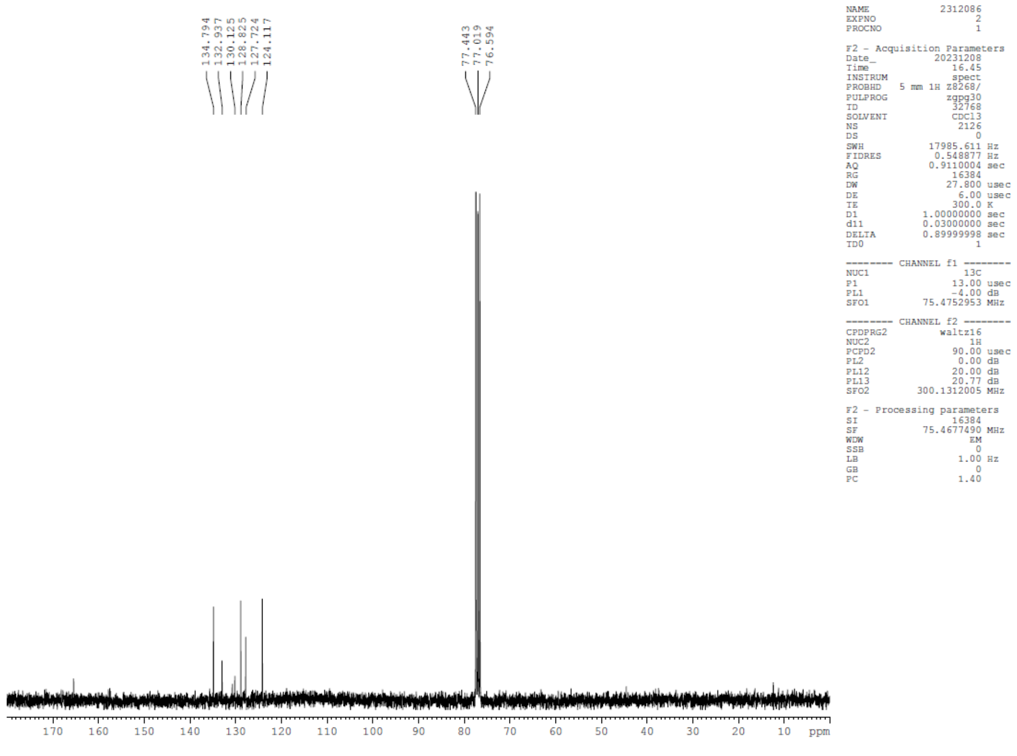

N-(1,3-dioxo-1,3-dihydro-2H-isoindol-2-yl)-4-methoxybenzamide (LD-F 02): Molecular formula: $C_8H_{10}N_2O_2$. Molar mass: 296.27 g/mol. Melting point (°C): 238; (theor. 240). FTIR (cm^{-1}): 1733.2 (C=O, imide); 1602.8 (N-H); 708.2 (C-H, aromatic ring). NMR - ^1H ($CDCl_3$) (ppm): 1.595 (chloroform water); 3.845 (CH_3); 7.240 (chloroform); 7.766 - 7.924 (aromatic ring); 8.226 (NH). NMR - ^{13}C ($CDCl_3$) (ppm): 55.488 (CH_3); 77.022 (chloroform); 114.025 - 134.728 (aromatic ring); 165.591 (C=O, imide).

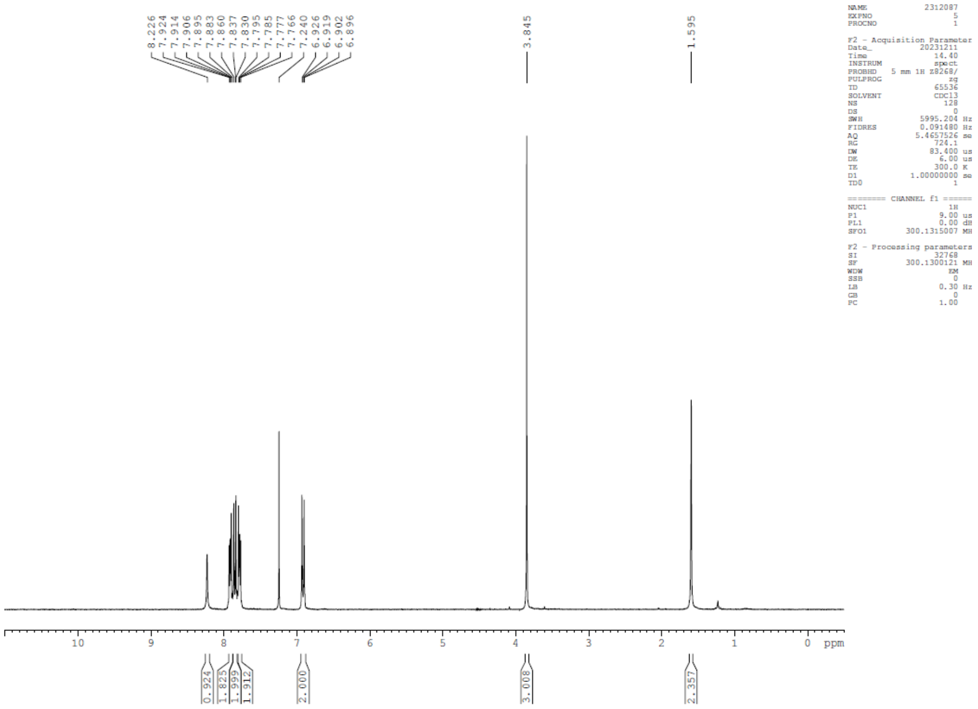

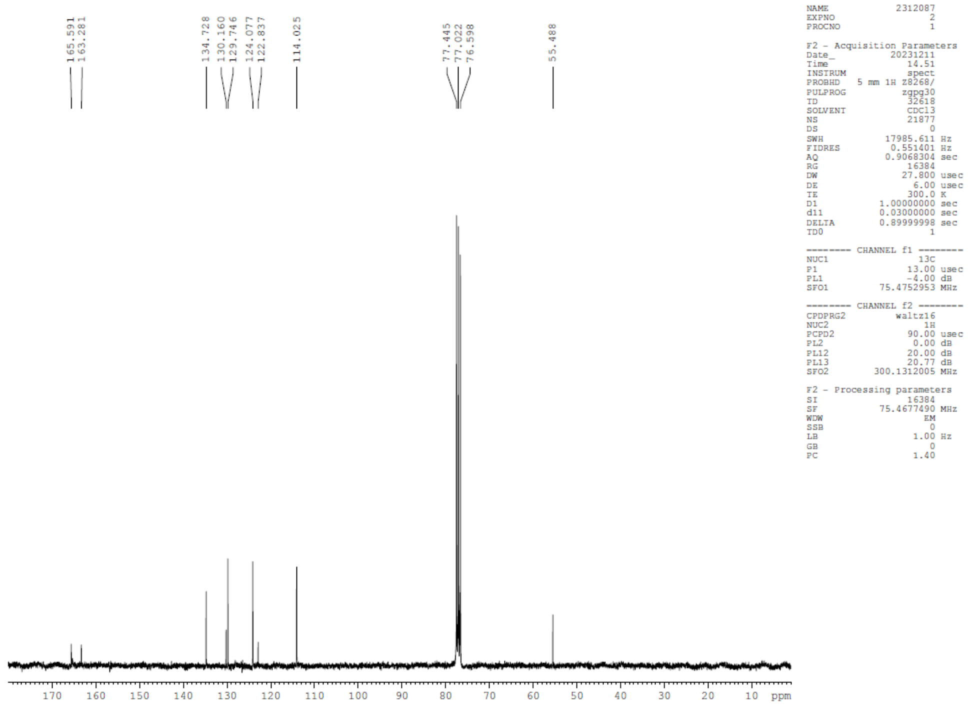

2-(1,3-thiazol-2-yl)-1H-isoindole-1,3(2H)-dione (LD-F 03): Molecular formula: $C_{11}H_6N_2O_2S_2$. Molar mass: 230.24 g/mol. Melting point (°C): 116 - 118; (theor. 117 - 118). FTIR (cm^{-1}): 1725.8 (C=O, imide); 1602.8 (N-H); 790.2 (C-S); 708.2 (C-H, aromatic ring). NMR - ^1H ($CDCl_3$) (ppm): 1.588 (chloroform water); 7.240 (chloroform); 7.304 - 7.991 (aromatic ring). NMR - ^{13}C ($CDCl_3$) (ppm): 77.021 (chloroform); 117.280 (C-S); 124.467 - 135.191 (aromatic ring); 165.659 (C=O, imide).

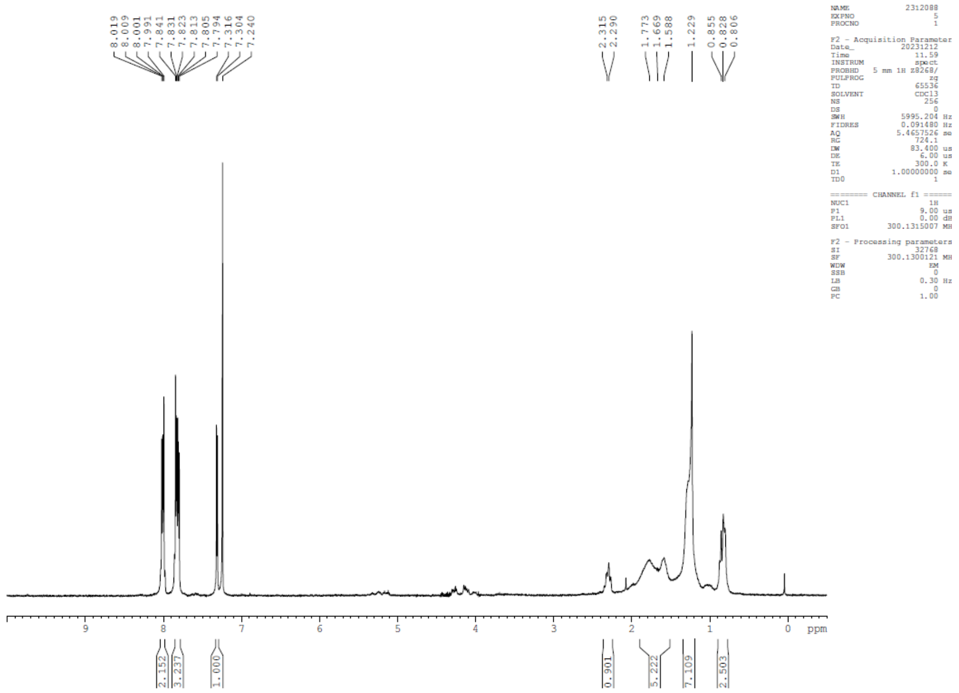

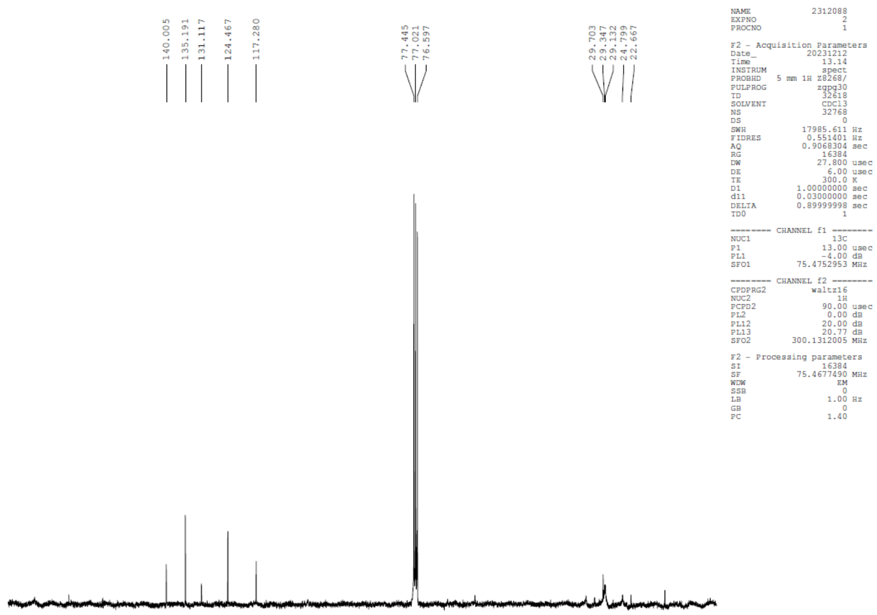

2-(1,3-benzothiazol-2-yl)-1H-isoindole-1,3(2H)-dione (LD-F 04): Molecular formula: $C_{15}H_8N_2O_2S$. Molar mass: 280.30 g/mol. Melting point (°C): 142 - 144; (theor. 143 - 147). FTIR (cm^{-1}): 1722.0 (C=O, imide); 1550.6 (N-H); 764.1 (C-S); 711.9 (C-H, aromatic ring). NMR - ^1H (DMSO) (ppm): 2.500 (DMSO); 3.344 (chloroform water); 7.302 - 8.172 (aromatic ring). NMR - ^{13}C (DMSO) (ppm): 39.233 (DMSO); 158.240 (C-S); 120.556 - 136.407 (aromatic ring); 167.769 (C=O, imide).

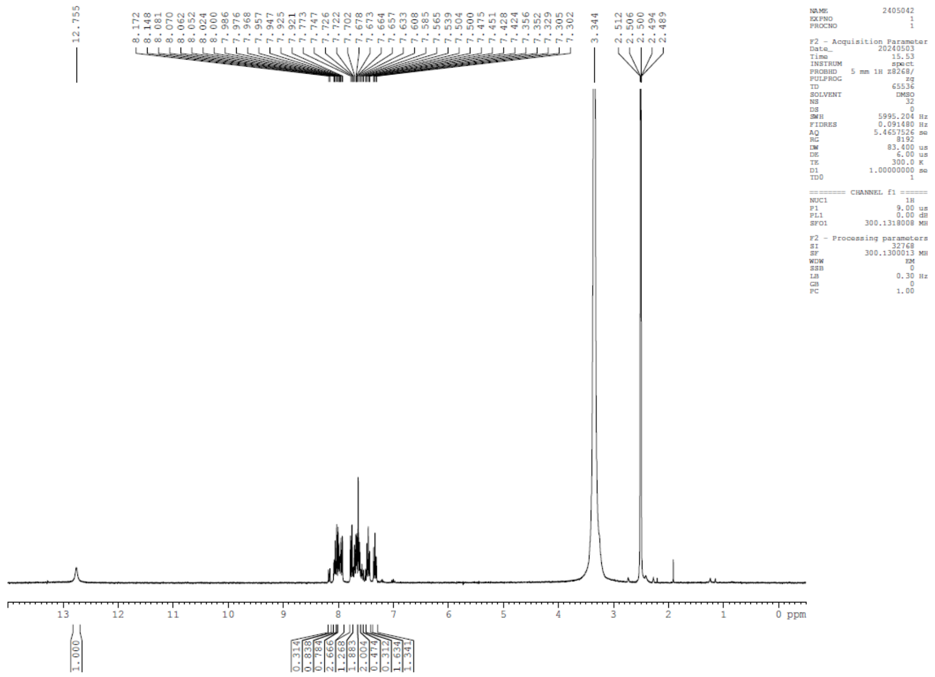

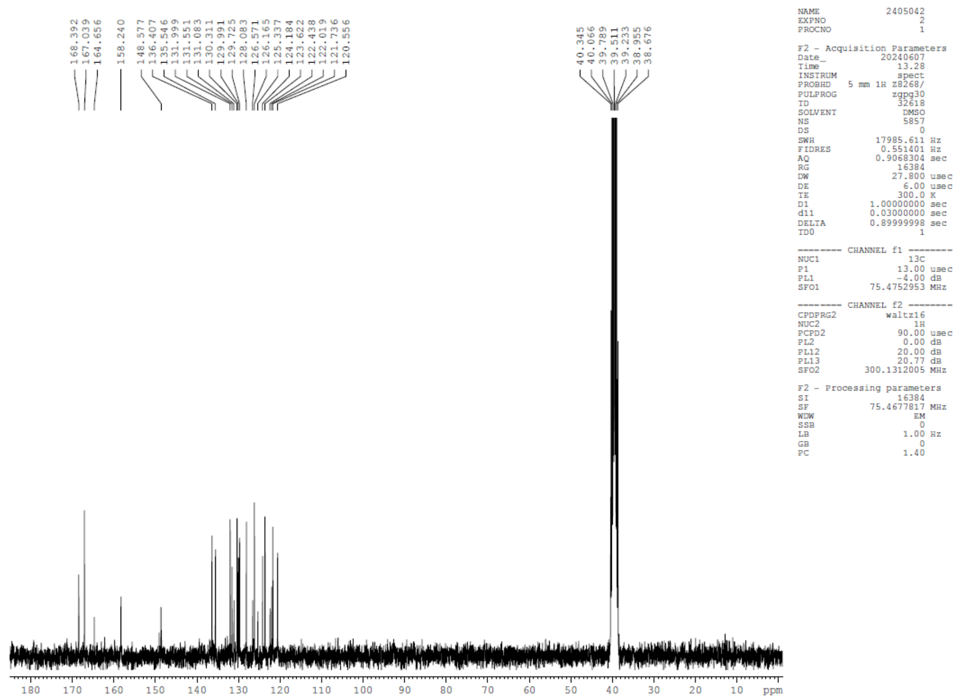

2-(4-methylphenyl)-1H-isoindole-1,3(2H)-dione (LD-F 05): Molecular formula: $C_{15}H_{11}NO_2$. Molar mass: 237.25 g/mol. Melting point (°C): 206; (theor. 206). FTIR (cm^{-1}): 1707.1 (C=O, imide); 1515.0 (N-H); 693.3 (C-H, aromatic ring). NMR - ^1H ($CDCl_3$) (ppm): 2.391 (CH_3); 7.240 (chloroform); 7.291 - 7.942 (aromatic ring). NMR - ^{13}C ($CDCl_3$) (ppm): 21.230 (CH_3); 77.050 (chloroform); 123.703 - 138.206 (aromatic ring); 167.458 (C=O, imide).

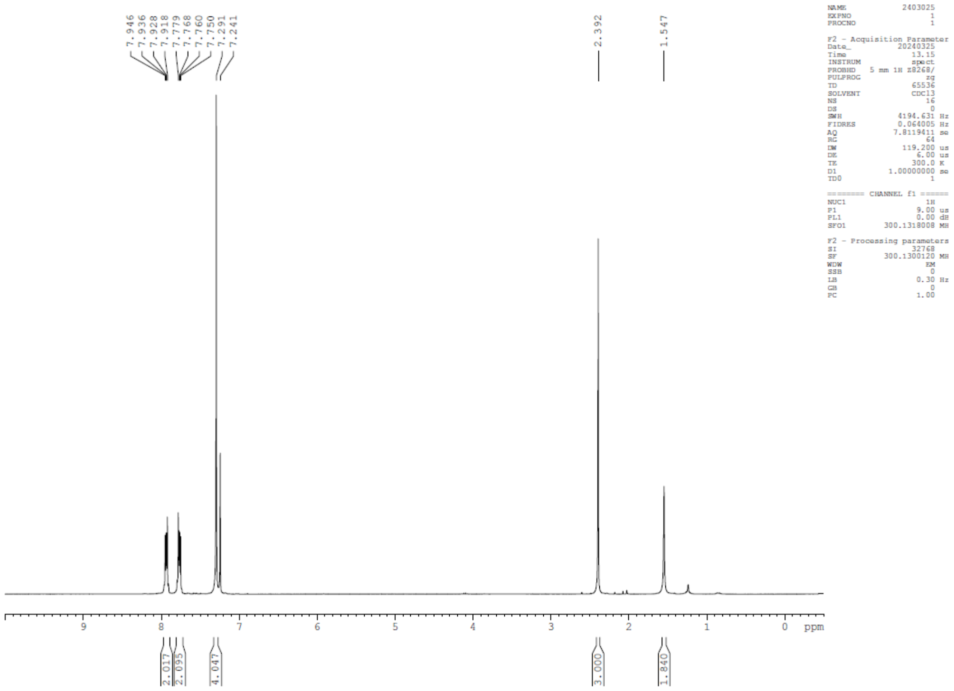

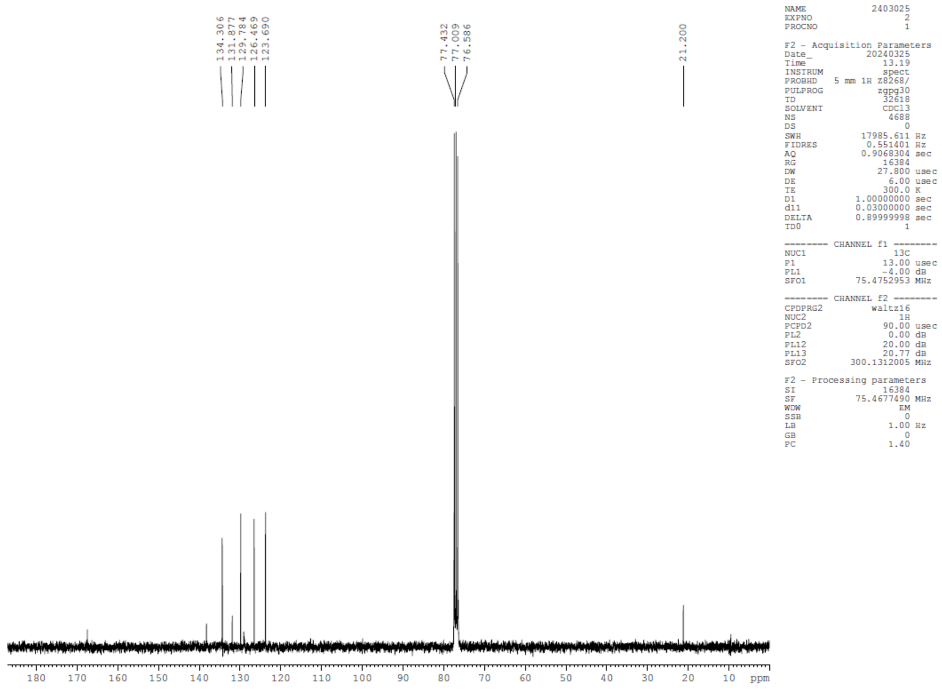

2-(3,5-dimethylphenyl)-1H-isoindole-1,3(2H)-dione (LD-F 06): Molecular formula: $C_{16}H_{13}NO_2$. Molar mass: 251.27 g/mol. Melting point (°C): 153 - 154; (theor. 154). FTIR (cm^{-1}): 1702.8 (C=O, imide); 711.9 (C-H, aromatic ring). NMR - ^{1}H ($CDCl_3$) (ppm): 2.357 (CH_3); 7.240 (chloroform); 7.000 - 7.914 (aromatic ring). NMR - ^{13}C ($CDCl_3$) (ppm): 21.288 (CH_3); 77.014 (chloroform); 123.673 - 138.904 (aromatic ring); 167.482 (C=O, imide).

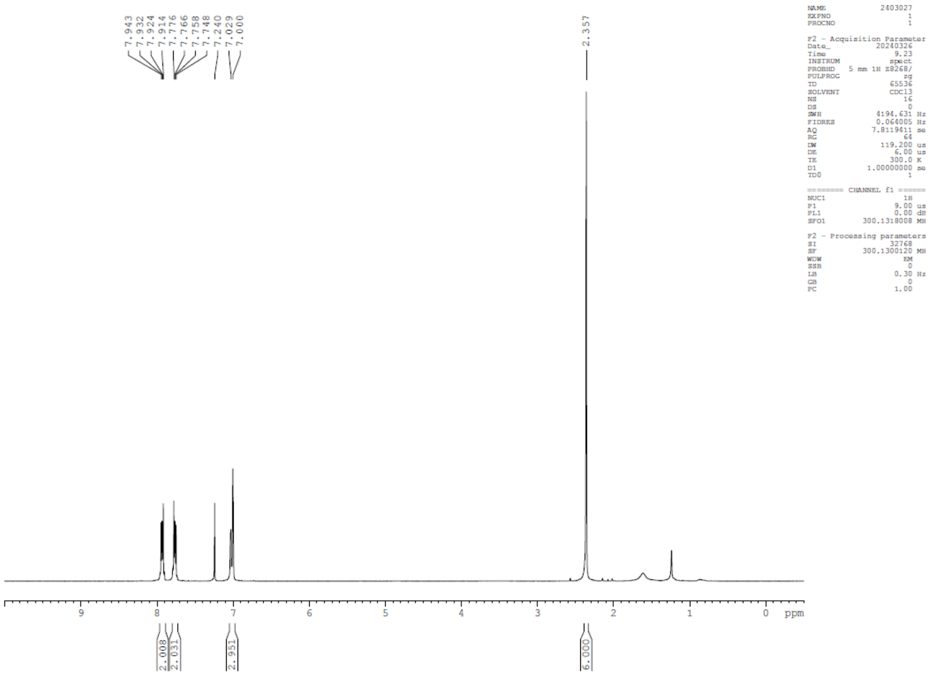

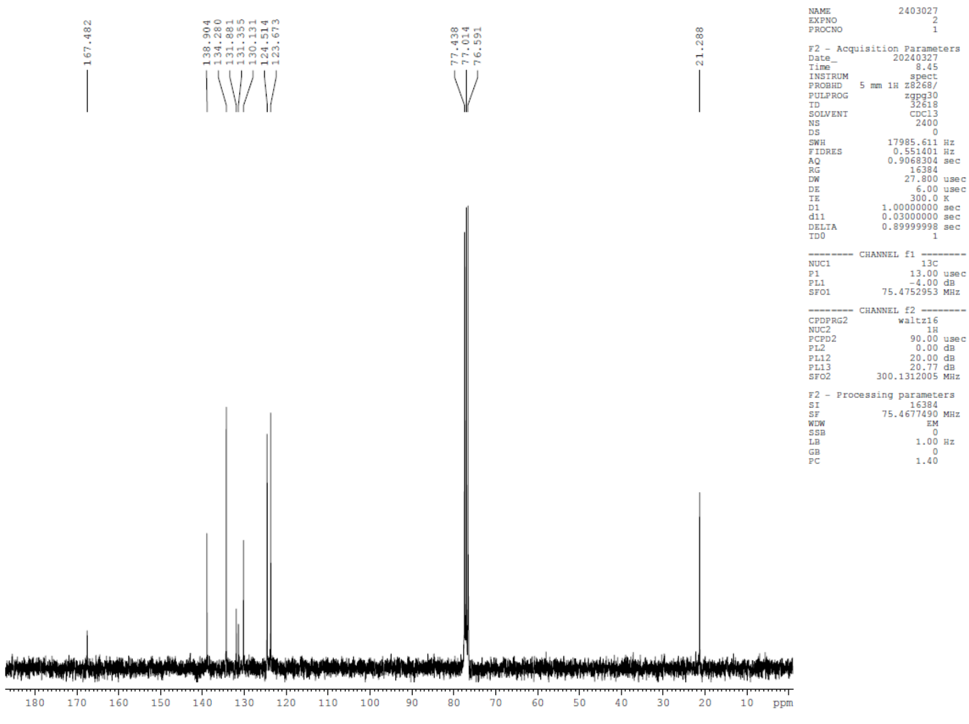

2-(4-ethylphenyl)-1H-isoindole-1,3(2H)-dione (LD-F 07): Molecular formula: $C_{16}H_{13}NO_2$. Molar mass: 251,27 g/mol. Melting point (°C): 176 - 178; (177 theor.). FTIR (cm^{-1}): 1703.4 (C=O, imide); 1610.2 (N-H); 711.9 (C-H, aromatic ring). NMR - ^1H ($CDCl_3$) (ppm): 1.283 - 2.730 (CH_3); 7.241 (chloroform); 7.317 - 7.949 (aromatic ring). NMR - ^{13}C ($CDCl_3$) (ppm): 15.394 - 28.595 (CH_3); 77.007 (chloroform); 123.690 -134.302 (aromatic ring); 167.137 (C=O, imide).

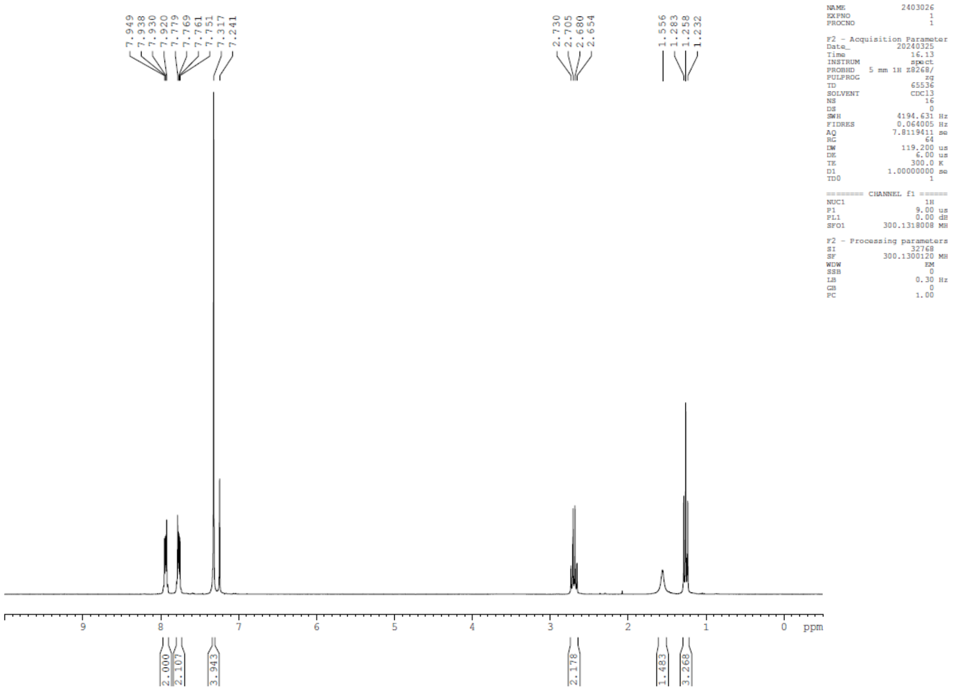

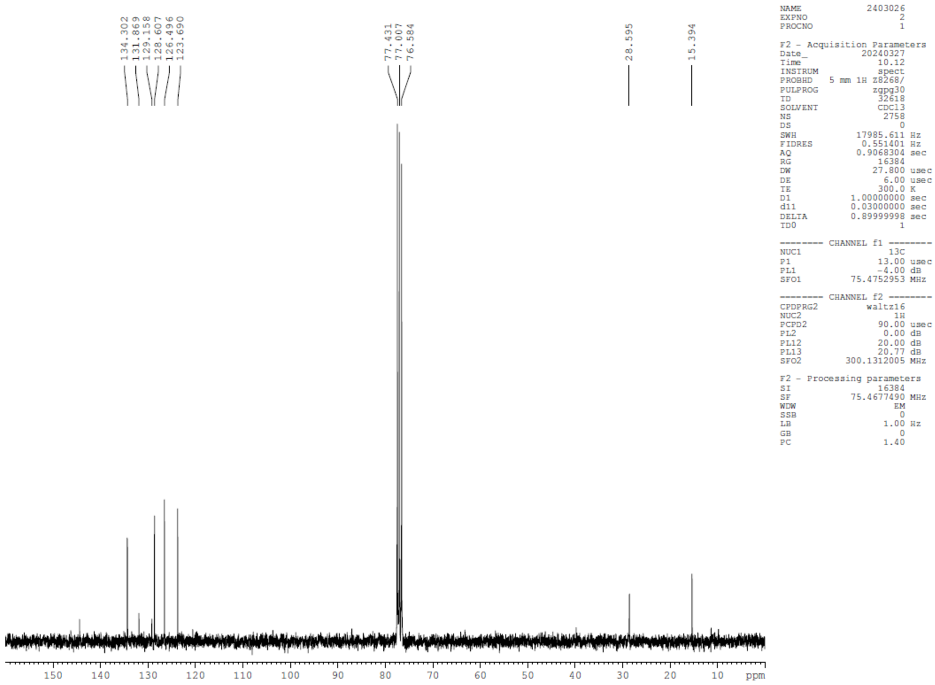

2-(2,4-dinitrophenyl)isoindole-1,3-dione (LD-F 08): Molecular formula: $C_{14}H_7N_3O_6$. Molar mass: 313.22 g/mol. Melting point (°C): 274 - 278; (theor. 274 -276). FTIR (cm^{-1}): 1729.5 (C=O, imide); 700.7 (C-H, aromatic ring). NMR - ^1H (DMSO) (ppm): 2.500 (DMSO); 3.349 (N-H); 7.571 – 8.931 (aromatic ring). NMR - ^{13}C ($CDCl_3$) (ppm): 39.516 (chloroform); 115.957 – 138.325 (aromatic ring); 147.332 (C-N); 165.430 (C=O, imide).

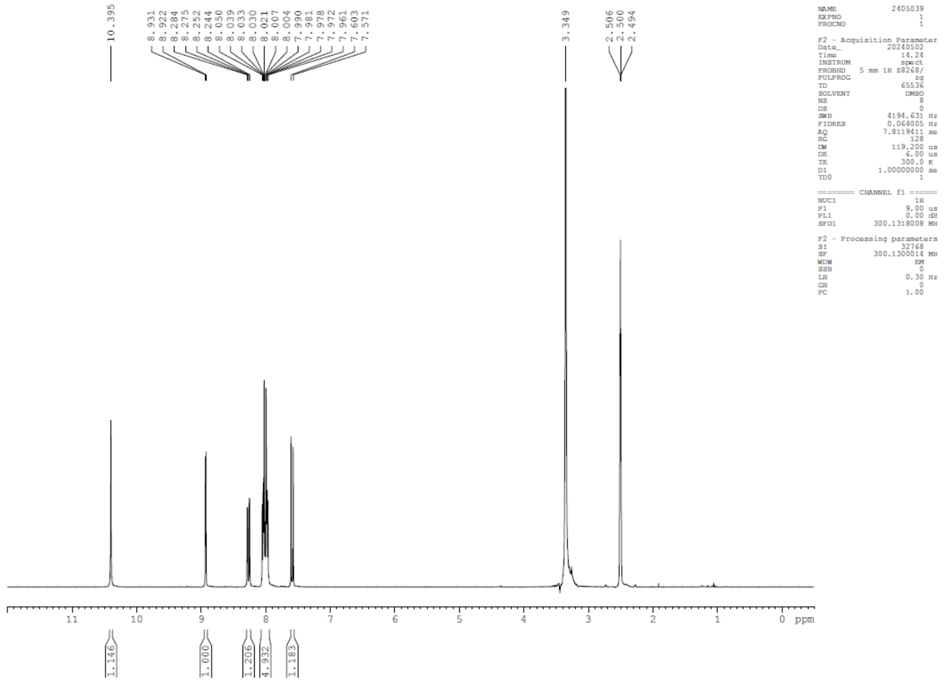

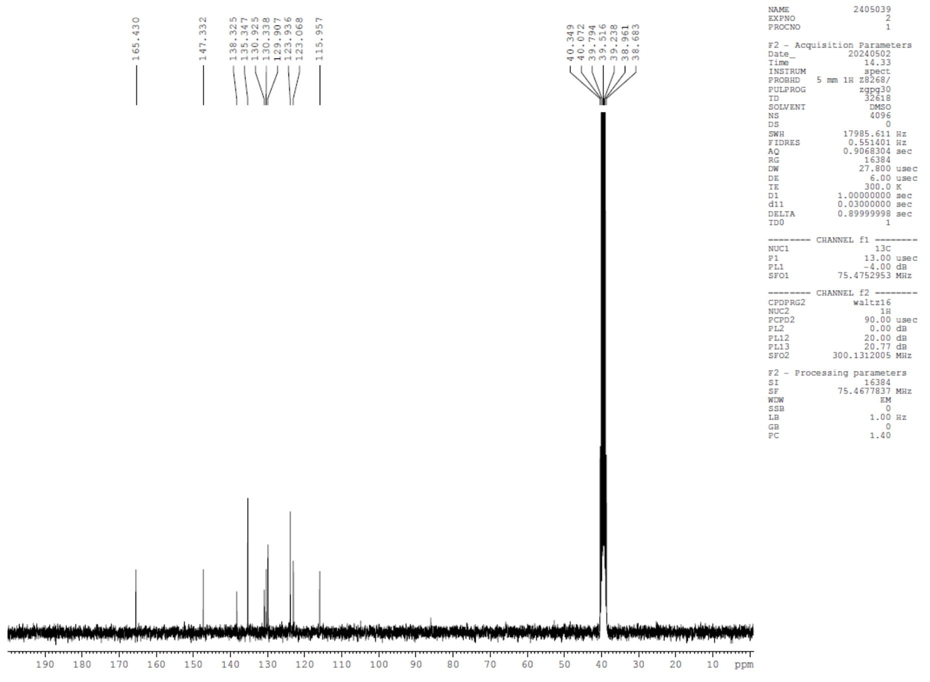

2-(pyridin-4-yl)-1H-isoindole-1,3(2H)-dione (LD-F 09): Molecular formula: $C_{13}H_8N_2O_2$. Molar mass: 224.21 g/mol. Melting point (°C): 233; (theor. 232 - 233). NMR - ^1H ($CDCl_3$) (ppm): 7.240 (chloroform); 7.640 - 7.855 (aromatic ring). NMR - ^{13}C ($CDCl_3$) (ppm): 77.005 (chloroform); 109.007 – 150.267 (aromatic ring); 167.122 (C=O, imide).

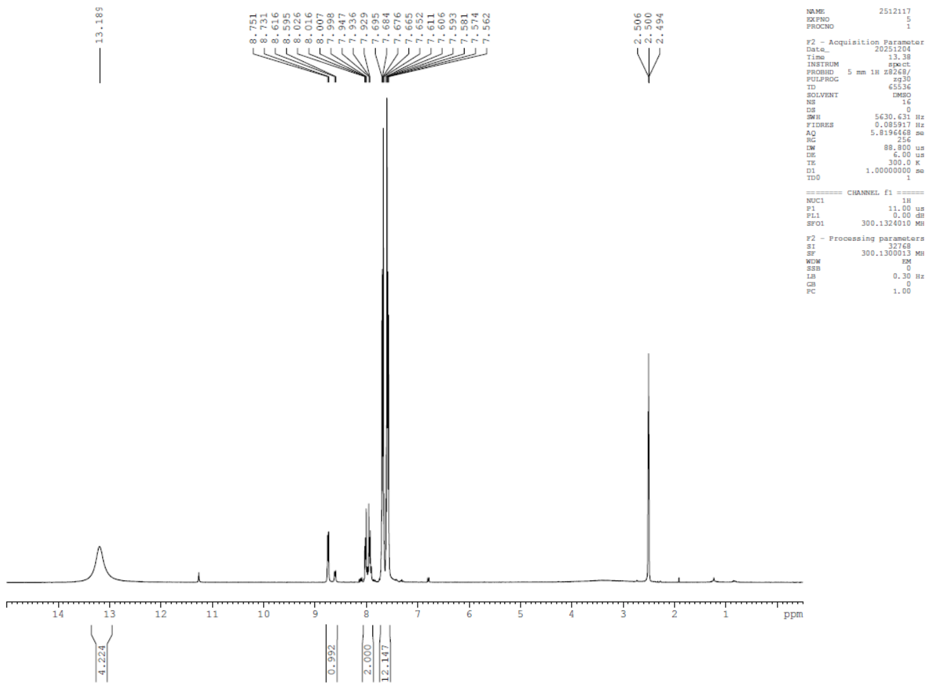

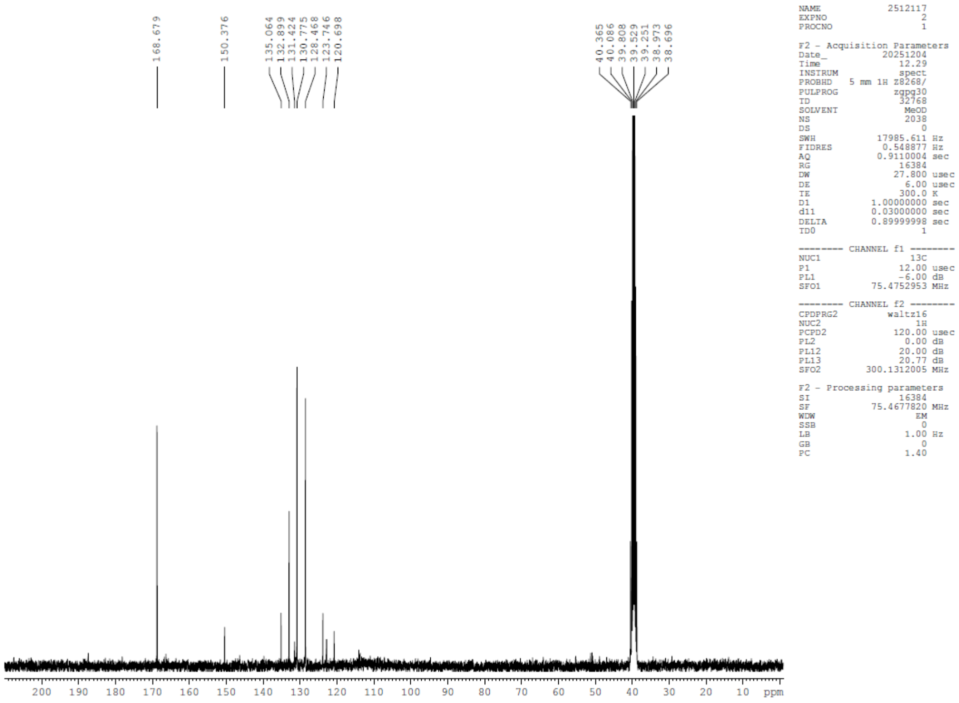

2-(4-methoxyphenyl)-1H-isoindole-1,3(2H)-dione (LD-F 10): Molecular formula: $C_{15}H_{11}NO_3$. Molar mass: 253.25 g/mol. Melting point (°C): 162; (theor. 162). FTIR (cm-1): 1707.1 (C=O, imide); 1610.2 (N-H); 708.2 (C-H, aromatic ring). NMR - ^1H ($CDCl_3$) (ppm): 3.833 (CH3); 6.989 - 7.941 (8H benzene). NMR - ^{13}C ($CDCl_3$) (ppm): 55.511 (CH3); 114.495 - 134.279 (12C benzene); 167.199 (C=O, imide).

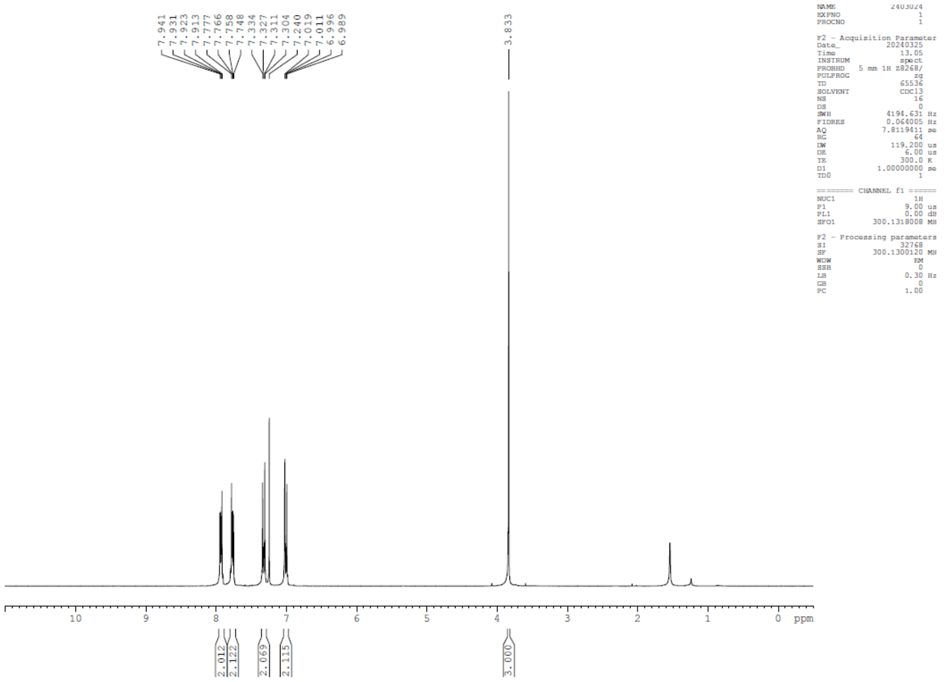

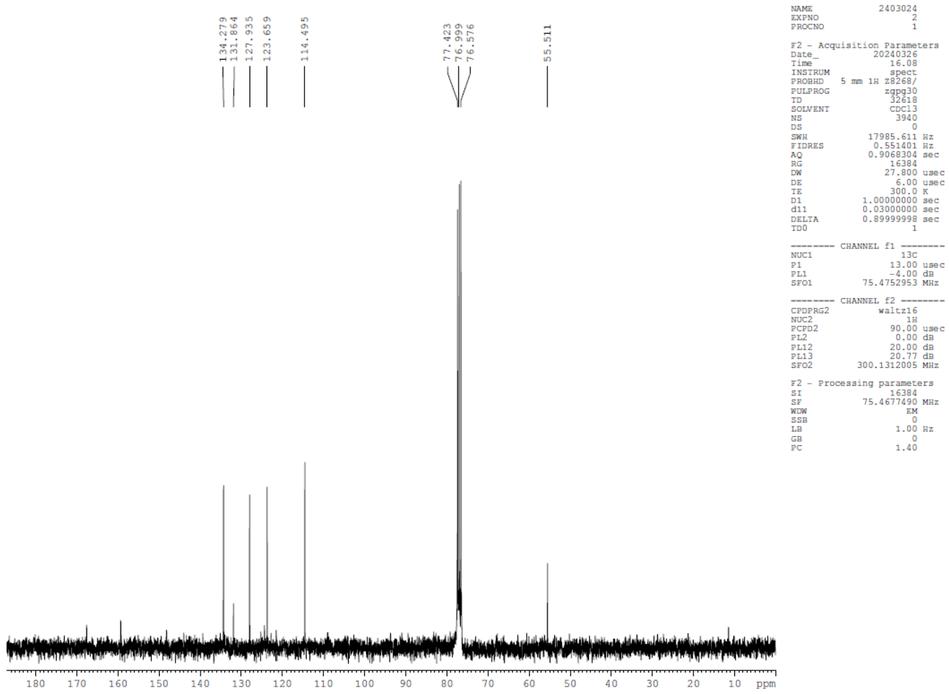

2-(4-acetylphenyl)-1H-isoindole-1,3(2H)-dione (LD-F 11): Molecular formula: $C_{16}H_{11}NO_3$. Molar mass: 265.26 g/mol. Melting point (°C): 238; (theor. 240). FTIR (cm-1): 1707.1 (C=O, imide); 1599.0 (N-H); 715.6 (C-H, aromatic ring). NMR - ^{1}H (DMSO) (ppm): 2.500 (CH3); 2.631 (DMSO); 7.621 - 8.121 (Aromatic ring). NMR - ^{13}C (DMSO) (ppm): 26.870 (CH3); 39.514 (DMSO); 123.596 - 134.901 (Aromatic ring); 166.688 (C=O, imide); 197.381 (C=O).

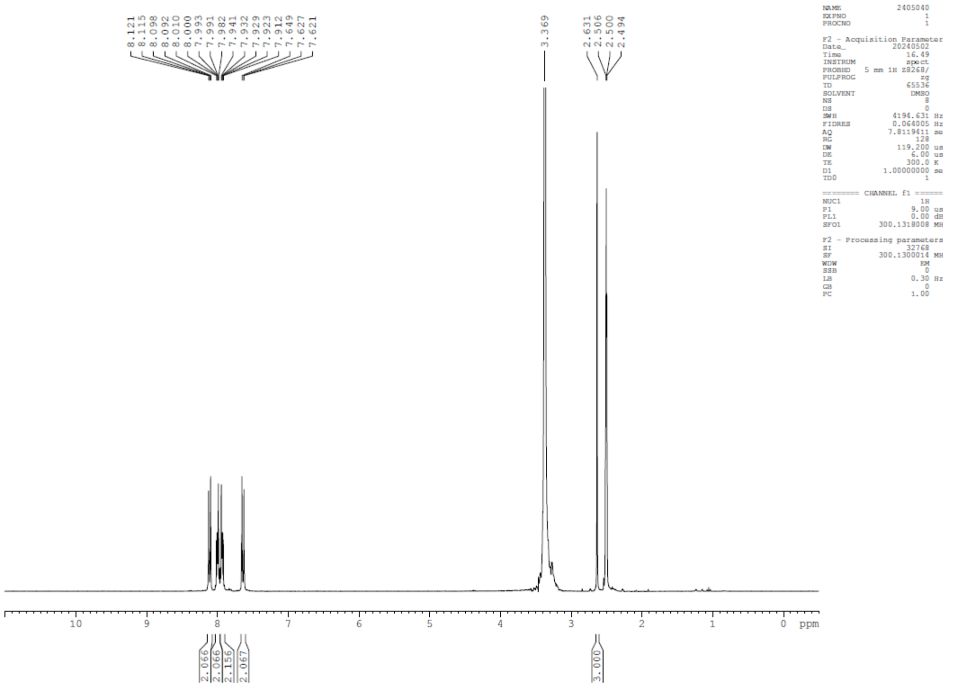

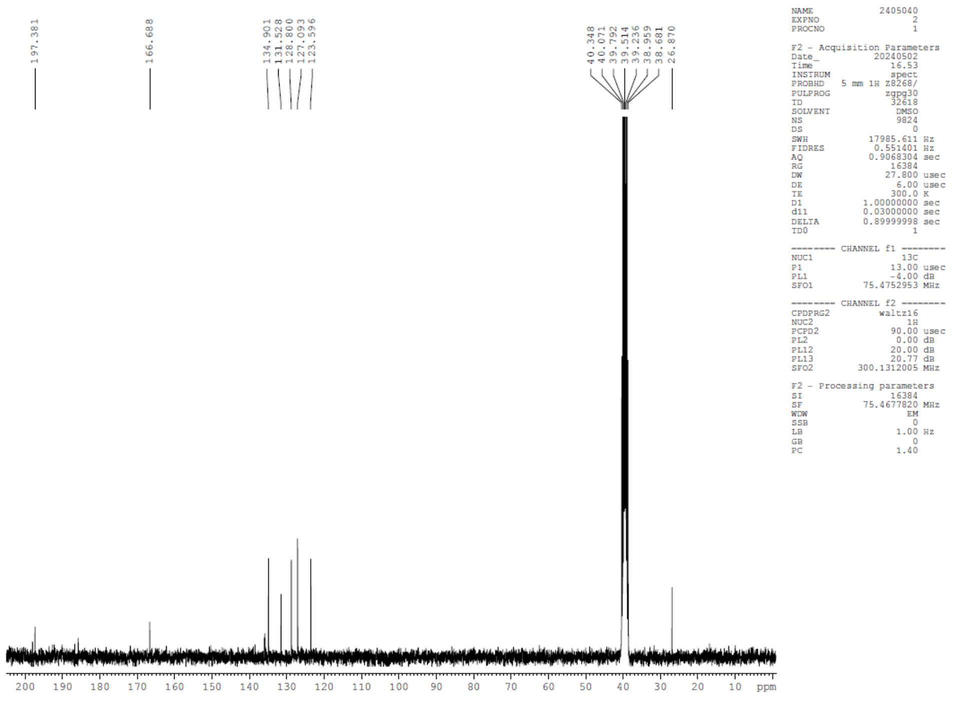

2-(phenylsulfanyl)-2,3-dihydro-1H-isoindole-1,3-dione (LD-F 12): Molecular formula: $C_{14}H_9NO_2S$. Molar mass: 255.29 g/mol. Melting point (°C): 205 D; (theor. 202 - 206 D). FTIR (cm-1): 1699.7 (C=O, imide); 1520.8 (N-H); 752.9 (C-S); 704.5 (C-H, aromatic ring). NMR - ^1H (DMSO) (ppm): 2.499 (DMSO); 3.362 (HS); 7.470 - 8.184 (Aromatic ring). NMR - ^{13}C (DMSO) (ppm): 39.519 (DMSO); 122.257 - 135.535 (Aromatic ring); 166.390 - 168.788 (C=O, imide).

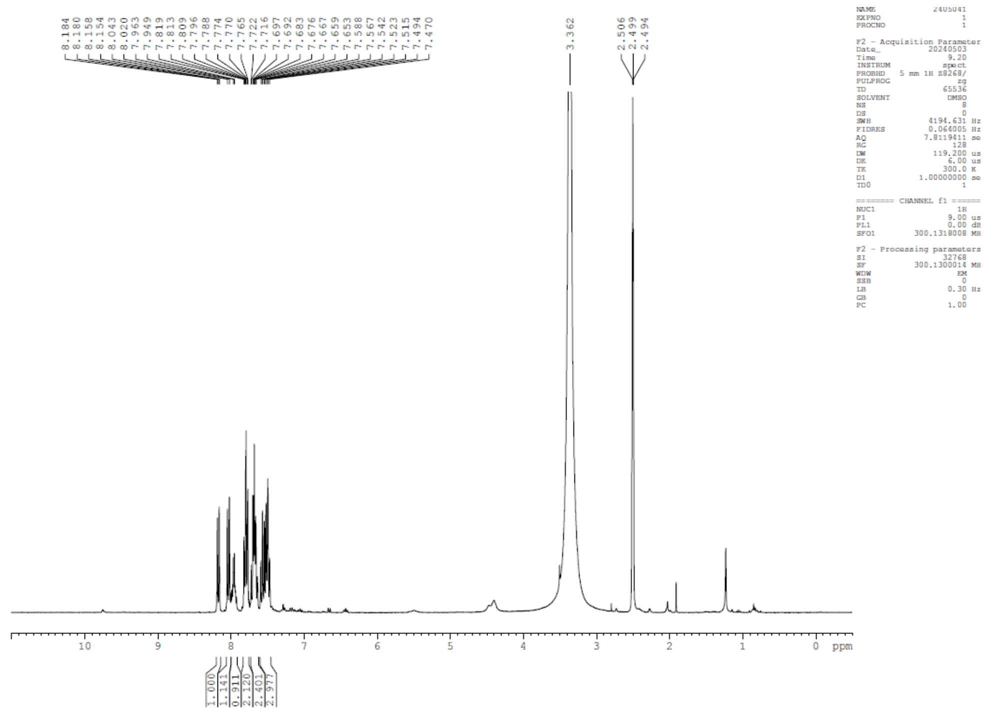

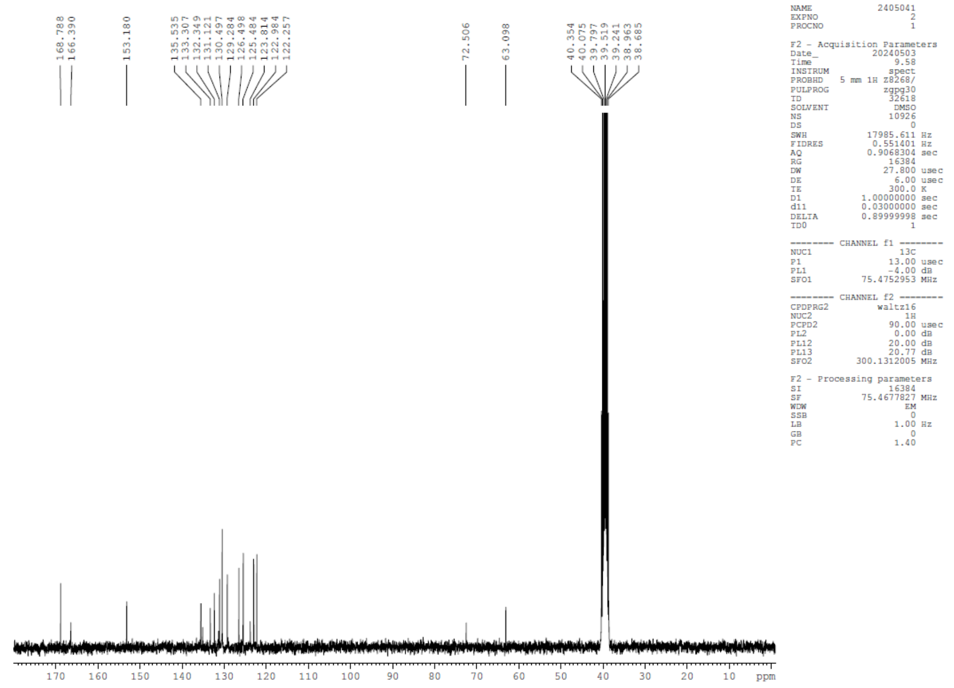

2-(2-phenylethyl)-1H-isoindole-1,3(2H)-dione (LD-F 13): Molecular formula: $C_{16}H_{13}NO_2$. Molar mass: 251,27 g/mol. Melting point (°C): 130 - 132; (131 - 132 teor.). FTIR (cm^{-1}): 1703.4 (C=O, imida); 752.9 (C-S); 708.2 (C-H, aromatic ring). RMN - ^{1}H (DMSO) (ppm): 2.501 (DMSO); 2.916 - 3.834 (CH_3); 7.171 - 7.829 (aromatic ring). RMN - ^{13}C (DMSO) (ppm): 39.501 (DMSO); 40.345 (CH_3); 123.068 - 138.269 (aromatic ring); 167.726 (C=O, imide).

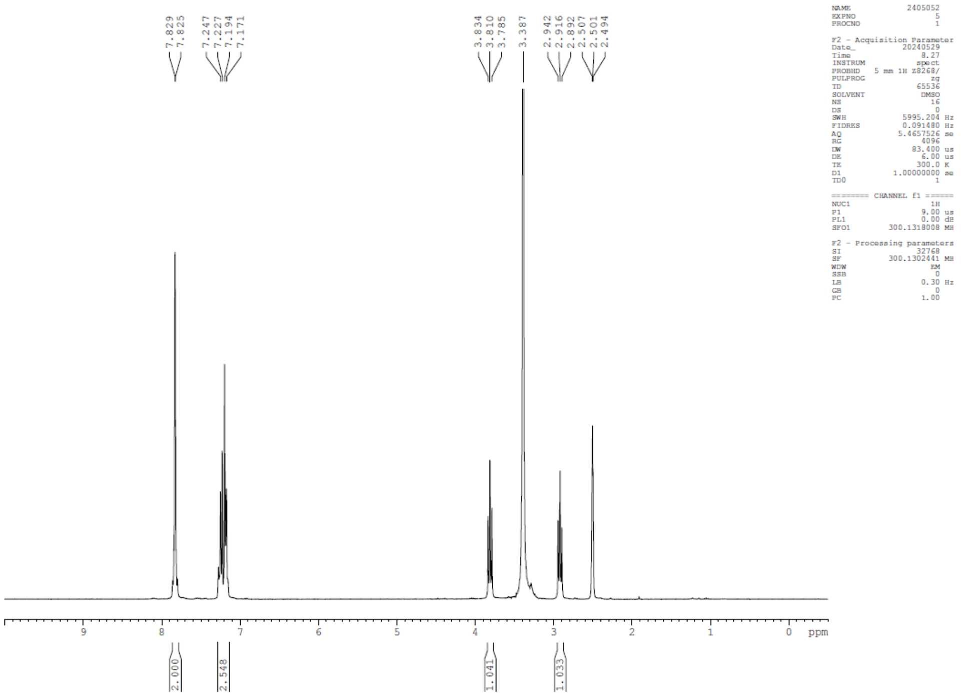

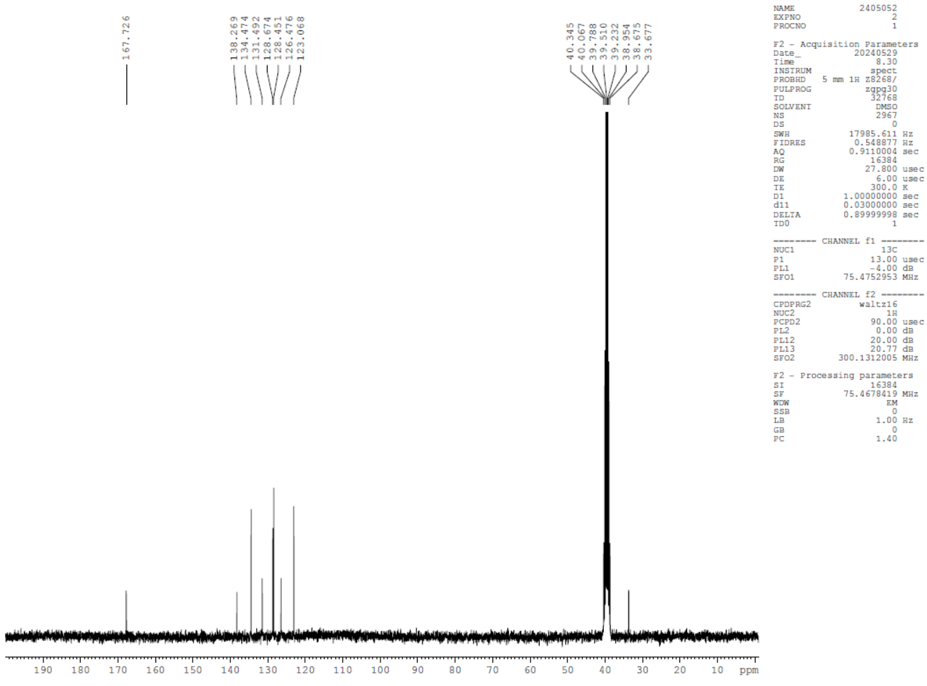

2-(4-chlorophenyl) -1H-isoindole-1,3(2H)-dione (LD-F 14): Molecular formula: $C_{14}H_8ClNO_2$. Molar mass: 257.67 g/mol. Melting point (°C): 180 - 184; (theor. 182 - 183). FTIR (cm-1): 1699.7 (C=O, imide); 752.9 (C-Cl); 715.6 (C-H, aromatic ring). NMR - ^{1}H (DMSO) (ppm): 2.501 (DMSO); 7.484 - 7.996 (aromatic ring). NMR - ^{13}C (DMSO) (ppm): 39.515 (DMSO); 123.515 - 134.805 (aromatic ring); 166.842 (C=O, imide).

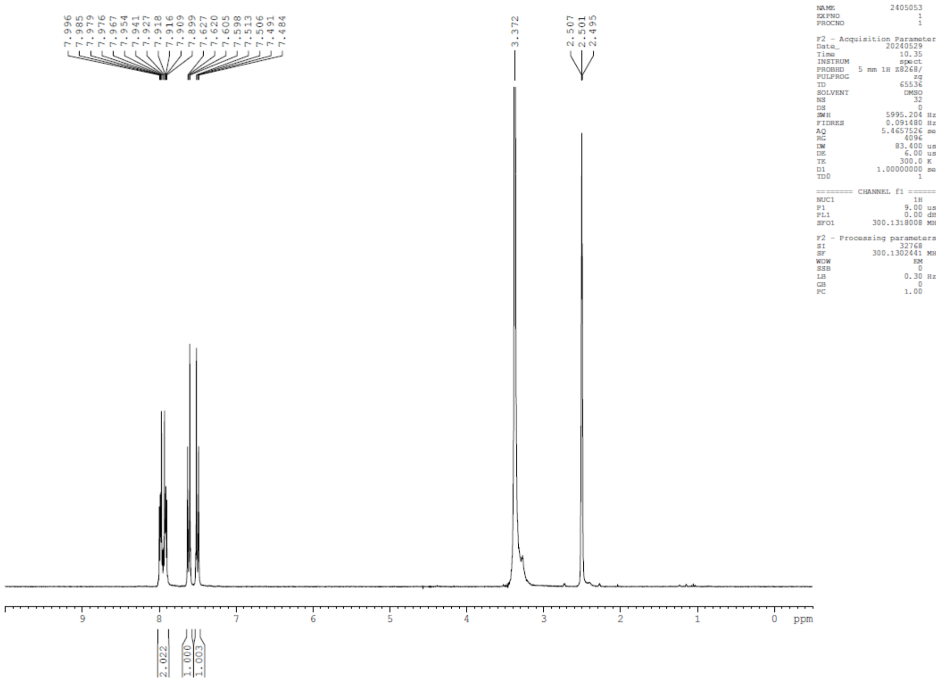

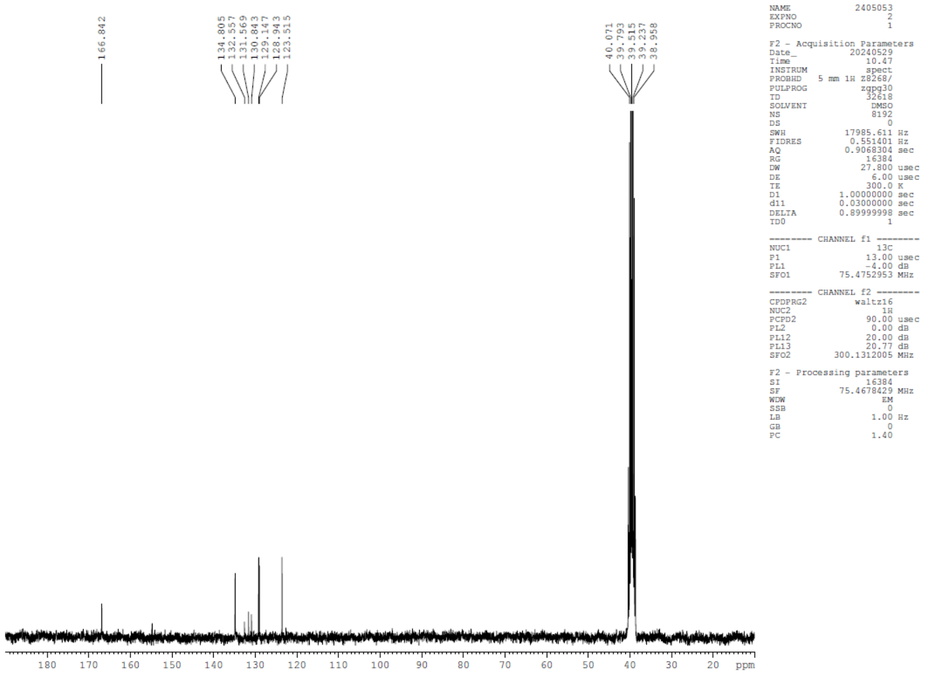

2-benzyl-1H-isoindole-1,3(2H)-dione (LD-F 15): Molecular formula: $C_{15}H_{11}NO_2$. Molar mass: 237.25 g/mol. Melting point (°C): 124; (theoretical 124). FTIR (cm-1): 793.9 (C-H, aromatic ring); 1707.8 (C=O, imide). NMR - ^{1}H (DMSO) (ppm): 2.501 (DMSO); 4.770 (CH3); 7.289 - 7.918 (Aromatic ring). NMR - ^{13}C (DMSO) (ppm): 39.512 (DMSO); 40.872 (CH3); 123.276 - 136.698 (aromatic ring); 167.769 (C=O, imide).

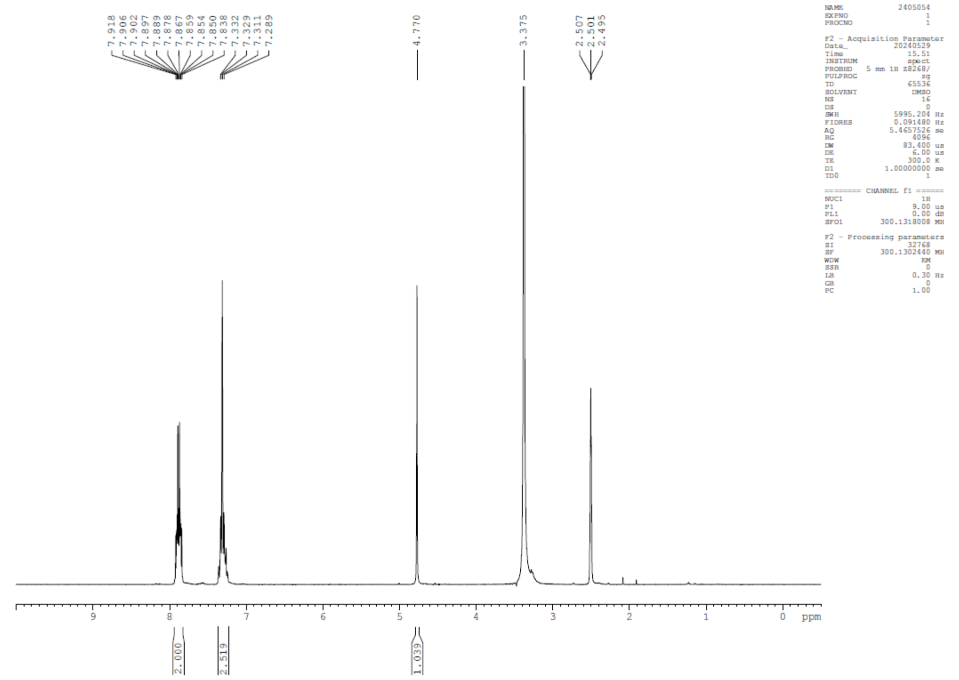

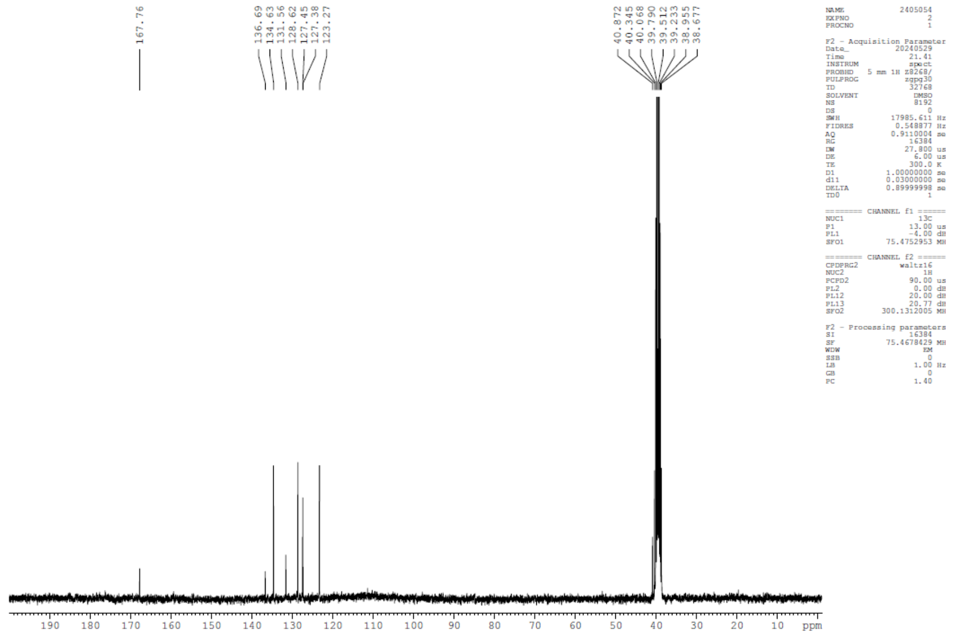

Supplement: Supplementary file 1 — Supporting File 1: cbdv70808‐sup‐0001‐SuppMat.pdf [file CBDV-23-e02823-s001.pdf]
